# Women’s experiences of a medically supervised fasting intervention for infertility: a qualitative study

**DOI:** 10.1186/s12905-026-04978-y

**Published:** 2026-10-09

**Authors:** Jocelyn N. Behling, Katharina T. May, Katharina Batschari, Katharina Sochiera-Plegniere, Sarah B. Blakeslee, Wiebke Stritter, Michael Jeitler, Farid I. Kandil, Barbara Kunz, Alexandra Prüß, Andreas Michalsen, Daniela A. Koppold

**Affiliations:** 1 Institute of Social Medicine, Epidemiology, and Health Economics, Charité – Universitätsmedizin Berlin, Corporate Member of Freie Universität Berlin and Humboldt-Universität zu Berlin, Charitéplatz 1, Berlin, 10117 Germany; 2 Department of Internal Medicine and Nature-Based Therapies, Immanuel Hospital Berlin, Am Kleinen Wannsee 5A, Berlin, 14109 Germany; 3 Charité Competence Center for Traditional and Integrative Medicine (CCCTIM), Berlin Institute of Health, Charité – Universitätsmedizin Berlin, Corporate Member of Freie Universität Berlin, Humboldt-Universität zu Berlin, Charitéplatz 1, Berlin, 10117 Germany

**Keywords:** Fasting, Infertility, Qualitative research, Personal narratives as topic, Motivation, Treatment acceptability

## Abstract

**Background:**

Infertility affects couples in Germany and worldwide, with impacts on quality of life. Although fertility treatments are available, success rates often remain limited, and treatments are associated with physical and psychological burdens. Consequently, many seek alternative or complementary approaches to support fertility. To investigate one such approach, an RCT was conducted to examine the effects of a medically supervised prolonged, fluid-only fasting intervention (< 500 kcal/day) on fertility-related outcomes. Nested within this trial, this qualitative study explores women’s experiences with the intervention, focusing on its acceptability and its perceived physical and psychological effects.

**Methods:**

The study accompanied a randomized controlled trial in which fasting served as an intervention to assess its potential effects on fertility. Semi-structured individual interviews were conducted after the intervention and analysed using inductive thematic analysis, with regular research group discussions ensuring consistency and rigor. All participants available and willing to participate in the qualitative study were interviewed. In total, eight women were interviewed between February 2022 and January 2023.

**Results:**

Fasting was generally well-received. Challenges included fatigue, taste dissatisfaction, and cravings, low mood and social withdrawal. Participants’ motivation, coping strategies, and dietitian support helped manage these difficulties. Reported benefits included physical well-being, improved mood, and a sense of inner balance. Fasting was valued for offering an active role in fertility treatment. Post-fast, some expressed a renewed hope for fertility treatment, while others described a shift away from their formerly intense focus on conception.

**Conclusions:**

Medically supervised prolonged fluid-only fasting was perceived as acceptable by women experiencing infertility, with both benefits and challenges reported throughout the fasting experience.

**Trial registration:**

Registry: ClinicalTrials.gov, TRN: NCT04942457, Registration date: 10 May 2021.

**Supplementary Information:**

The online version contains supplementary material available at https://doi.org/10.1186/s12905-026-04978-y.

## Introduction

Around 17.5% of couples worldwide suffer from infertility, defined as the inability to conceive after one year of unprotected intercourse [1].

Although the decision whether or not to have a child can be understood as an individual choice, it is at the same time embedded in societal expectations [2]. The emergence of the desire to have children can therefore also be understood within a social context in which parenthood is expected and associated with fulfilment and a desirable life [2]. This pronatalist normativity is reflected in various social norms and expectations, including gender and family norms as well as expectations within one’s social environment [2–4]. Furthermore, political and institutional frameworks can contribute to presenting parenthood as a desirable and taken-for-granted part of life [5]. “Having children” may therefore be part of a socially expected life course and linked to notions of a successful transition to adulthood, personal fulfilment, and a meaningful life [2, 6–8]. The development of a personal desire to have children thus occurs within a tension between individual life plans and societal expectations. An unfulfilled desire to have children can therefore be understood as a disruption to an individual’s life plans [8–10]. That can call previously taken-for-granted notions of identity, biography, and the future into question [11]. In the context of an unfulfilled desire for children, this experience may be accompanied by feelings of uncertainty, lack of control, social isolation, or a sense of failure [9, 12–14]. The unfulfilled desire for children, a description explicitly chosen to describe the wider impacts, disruptions and dimensions of infertility, can therefore significantly impact a couple’s quality of life and impose a serious psychological and financial burden [12, 13, 15]. At the same time, those affected find themselves involuntarily confronted with a deviation from a socially expected life course [16]. Infertility should therefore not be understood solely as an individual medical problem, but also as a social and biographical experience shaped by societal expectations regarding parenthood and family formation [3].

Although childlessness has long been socially and culturally problematized, its medicalisation has introduced new ways of defining and addressing difficulties in conceiving [3, 17, 18]. In this context, couples experiencing an unfulfilled desire for children may seek infertility treatment [19]. Although male factors contribute wholly or in part to 45.1% of infertility cases, current infertility treatments predominantly involve medical interventions for women, placing a greater share of the medical burden on them [20]. This occurs against the backdrop of longstanding gendered expectations surrounding reproduction, which have often placed greater responsibility for childbearing on women [18]. These assisted reproductive technologies (ART) are associated with several physical and psychological side effects, including multiple pregnancies, ovarian hyperstimulation syndrome, anxiety, and depression [20, 21]. Dropout rates increase from 26.5% after the first complete cycle to 47.9% after the fifth, with stress cited as common reason for discontinuation [22]. Furthermore, ART can impose a substantial financial burden, contributing to inequalities in access to fertility treatment [23]. In Germany, only 15% to 22% of treatment cycles result in a live birth, underscoring the need for supportive treatment options in fertility care [24]. These limitations and burdens highlight the need to consider supportive approaches that may complement conventional fertility care.

Taking aside anatomical abnormalities, in many cases reproductive dysfunction and infertility are connected to the dysfunction of certain physical metabolic, endocrine, inflammatory, and immune processes [25, 26]. Chronic low-grade inflammation, for example, has been implicated in impaired ovarian function and other reproductive processes relevant to fertility [25, 27]. Fasting has shown anti-inflammatory effects in clinical and experimental studies and may positively influence oxidative stress and metabolic parameters [28, 29]. Emerging evidence, particularly from studies in women with polycystic ovary syndrome (PCOS), further suggests that intermittent fasting may improve metabolic parameters and hormonal balance [30, 31].

Beyond its potential metabolic and reproductive effects, prolonged fasting may also affect psychological well-being. Previous studies have reported improvements in quality of life and mood following prolonged fasting, which may be particularly relevant given the substantial psychological burden associated with infertility [32–34]. Furthermore, women experiencing infertility frequently seek additional approaches to improve their chances of conception, enhance quality of life, and regain a sense of control over their bodies, and have expressed interest in integrating complementary approaches in addition to conventional fertility treatment [35, 36]. Available evidence suggests that prolonged fasting can be safely undertaken in appropriately selected individuals [32, 37]. However, little is known about how women experiencing infertility perceive and experience prolonged fasting, including its potential benefits, challenges, and acceptability. Against this background and as part of a broader investigation of prolonged fasting as a potential supportive treatment for women experiencing infertility, this qualitative study was conducted with the aim of gaining a more comprehensive understanding of their experiences. It aimed to explore the acceptability of prolonged fasting, with a particular focus on participants’ perceived physical and psychological effects. Furthermore, we explored participants’ needs, challenges, and resources to provide a more comprehensive understanding of their experiences and to identify aspects that may inform the further development and implementation of fasting as a supportive treatment [38, 39].

## Methods

Research steps and methodology for this qualitative study are reported with the COREQ checklist, presented in an Additional file (see Additional file 1) [40]. The study protocol was approved by the ethics committee of Charité-Universitätsmedizin Berlin in January 2018 (ID: EA4/216/17). It adheres to the Declaration of Helsinki criteria and Good Clinical Practice guidelines.

### Study design

This qualitative study accompanied an RCT investigating a medically supervised outpatient fasting intervention for women experiencing infertility. A CONSORT checklist for the RCT is provided in an additional file (see Additional file 2). The intervention consisted of a prolonged fluid-only fasting protocol of seven days with an option to continue fasting for up to 10 days [41]. The exact fasting duration was determined by the participants themselves in communication with the study physician and dieticians. During the fasting period, participants consumed up to 300 kcal per day in the form of vegetable juices and broths (“fasting meals”). A daily fluid intake of two to three litres was recommended (water / herbal tea). Details of the intervention are available in the “Handout Fasting” provided to participants and published as part of the study protocol of a similar intervention (online supplemental file 2: pages 4–16) [42]. Support during the fasting period also included regular telephone consultations with a dietitian (at least once before and twice during fasting), with additional support available as needed. Adherence to the fasting protocol was assessed during these phone calls, through questionnaires and at the visit with the study physician after the intervention. Semi-structured qualitative interviews were conducted after fasting completion (JNB). The interview guide was developed based on Helfferich’s framework, reviewed by experienced researchers, and pilot-tested (JNB, SB, WS, DAK). An additional file shows the interview guide in detail (see Additional file 3) [43]. The RCT and this accompanying trial were registered at ClinicalTrials.gov (NCT04942457). The results of the RCT are intended to be published elsewhere.

### Sampling and recruitment

**Fig. 1:**
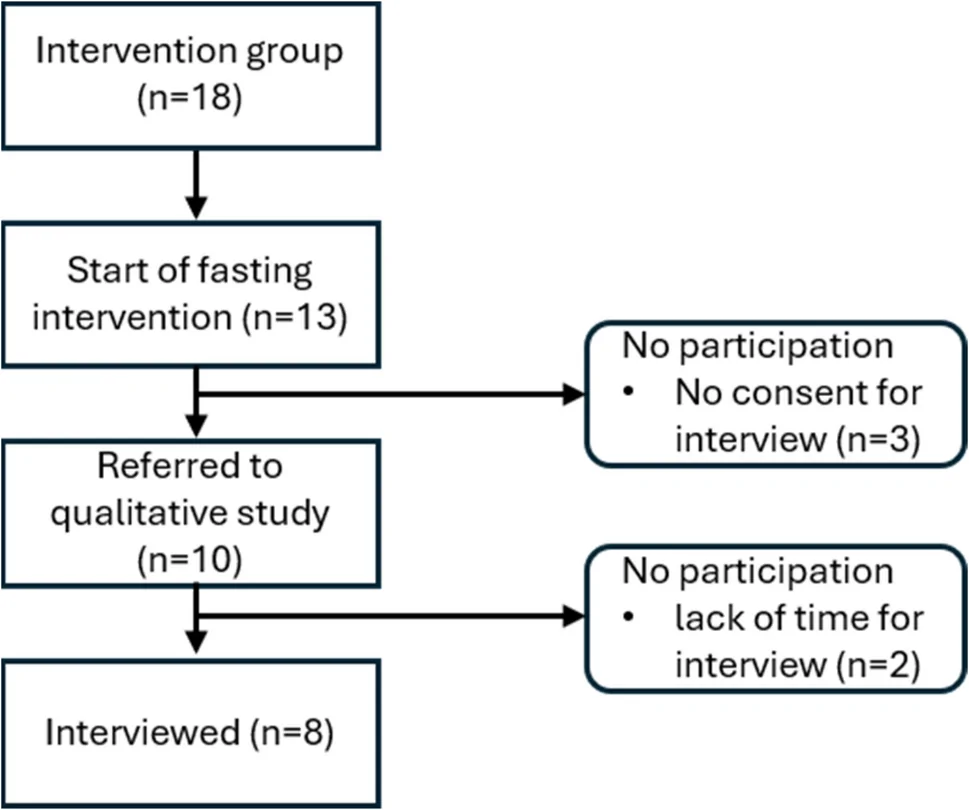
Study flow diagram

The participants of the RCT were recruited between December 2021 and December 2022 at three fertility centres in Berlin, Germany. After experiencing recruitment difficulties, recruitment was expanded to include social media platforms like Instagram and fertility-related websites. Women experiencing infertility aged 25–45 years with a BMI of 20–40 kg/m² were included unless they met one of the exclusion criteria presented in Table 1 and consequently randomized. Eighteen women were randomized to the intervention group of the RCT, of whom 13 started the intervention. All were invited to participate in a qualitative interview through written informed consent forms obtained prior to study participation; 10 subsequently consented to be contacted for the qualitative study. They were contacted by telephone by a female medical student with no prior relationship to the participants, received additional information about the interview, and provided renewed oral consent (JNB). Eight participants were ultimately interviewed (Fig. 1).

Initially, the interview sampling aimed to include a broad range of fasting experiences using maximum variation sampling and targeted theoretical saturation [44]. However, given the limited sample size resulting from recruitment challenges in the larger RCT and its premature closure, all eligible participants were approached for participation in the qualitative study, representing a pragmatic adaptation of the sampling strategy. Therefore, the initially planned target sample size for the qualitative study could not be achieved, and data collection could not be continued until theoretical saturation was reached.

**Table 1:** Inclusion and exclusion criteria

Inclusion criteria	Exclusion criteria
Female sex	Organic urogenital anomalies
Infertility	Severe medical conditions
Age 25–45 years	Past or present eating disorders
BMI 20–40 kg/m²	Inadequate German language skills
	Lack of internet access
	No consent to randomization
	Participation in other studies

### Data collection

Individual interviews were conducted in German, using the online platform Samedi, between February 2022 and January 2023. The interviews were scheduled for approximately an hour within five weeks after the fasting experience to ensure memory recall (JNB).

Notes were taken during and after the interviews to aid the interpretation of the data, and the interview guide was iteratively adapted. The interviews were audio recorded and then transcribed verbatim, controlled, and pseudonymized following the content semantic transcription rules described by Dresing and Pehl (JNB) [45].

Interviewed participants did not review their individual interview transcripts, nor were they personally involved in the analysis of their data.

### Data analysis

The analysis was based on Braun and Clarke’s method of thematic analysis and used MAXQDA 2022 (VERBI Software, 2021) for data analysis [46, 47]. Prior to coding, the transcripts were read, and first impressions of each interview were recorded. These impressions, along with post-interview notes, were used to write a case summary for each interview to provide a general overview of the different types of experiences described by fasting participants. This process allowed for recording the individual fasting contexts and their possible connection to the fasting experience. Next, the three most information-rich interviews were inductively coded to develop the initial category system. The resulting codes were sorted thematically, and content-based category definitions were formulated. The remaining interviews were coded inductively, either fitting into existing categories, prompting adaptations, or leading to new categories. This process and initial coding was conducted by JNB.

Emerging findings and categories were critically discussed with the research team and peer researchers to enhance the rigor and credibility of the analysis. This created a triangulation of investigator groups whereby preliminary findings were discussed at several stages of the analytic process. This was done with senior researchers (DAK, WS, SB) and in a regularly scheduled qualitative research colloquium with peer researchers (KTM, KB, KSP) in addition to the first author. Particular attention was paid to challenging or ambiguous passages and to the development and refinement of categories. Feedback from these discussions was incorporated into the analysis and used to review and refine the coding and categorization for consistency.

## Results

### Description of the sample

Of the 13 potential participants approached, eight agreed to and partook in an interview. Interview ranged in length from 37 to 68 min and had an average duration of 53 min. Participants’ ages were fairly evenly distributed, ranging from 30 to 44 years of age. All but one had university degrees, one had completed vocational training. Most participants were either partially or fully employed, while one was on extended medical leave and another was currently unemployed. Two participants had children. Participants differed in their previous and planned use of fertility treatment: some had already undergone several ART cycles and did not plan to pursue further treatment, some had decided against ART altogether, while others were planning or considering starting ART but remained uncertain about this step. For all but two participants, this intervention represented their first fasting experience. Those with previous fasting experience are noted and exemplified in the quotation descriptions (“2nd fasting”). All but one interviewed participant completed a seven-day fast, which was the minimum fasting duration specified in the study protocol. One participant ended her fast prematurely after six days.

### Description of positive fasting experiences

All participants frequently reported positive experiences during fasting. Positive emotions and mental states included a sense of well-being, improved mood, mental alertness, inner balance, calmness, and the mindful enjoyment of the simple fasting meals. Many participants also described feeling proud of a changed approach toward listening to and caring for their bodies through fasting. Participants described a changed sense of physical well-being, including feelings of lightness, physical alertness, and increased energy: *“And I really had*,* like*,* a razor-sharp mind. I didn’t forget anything. I got everything done that I wanted to. I was very fast*,* very clear in my communication.”* (Participant 3, aged 44, 2nd fasting, Pos.52).

The adoption of beneficial fasting practices and intentional slowing of routines were cited as factors contributing to these positive experiences. Fasting was perceived as an opportunity to incorporate moments of peace, physical activity, and self-care, which were facilitated by the elimination of regular mealtimes. In addition, maintaining or returning to previously beneficial routines, particularly sharing meals with their partners, was described as a source of well-being and was often more strongly appreciated in the context of increased mental alertness and inner balance.

*“But during that time*,* I just took everything more calmly. […] A bit of (pause) going inward*,* reflecting*,* you know?”* (Participant 8, aged 39, 2nd fasting, Pos.90).

*“I was much more relaxed than usual. Also much more balanced. […] It’s often the case that my mood fluctuates a lot or I react strongly to things that annoy me. And that was much more balanced.”* (Participant 7, aged 39, 1st fasting, Pos.24).

Beyond a gained sense of well-being, some participants reported improvements in pre-existing health issues or common complaints during the fasting period, such as hip pain or digestive problems.

Furthermore, some participants reported feelings of relief because anticipated fasting related challenges, such as concerns about low energy levels or hunger, did not materialize or because previous fasting-related difficulties were successfully overcome. Positive fasting experiences are also presented in an Additional file (see Additional file 4).

### Effects of fasting on attitudes towards wanting children

Many participants found fasting appealing as it allowed them to take an active role in their fertility treatment, describing it as an escape from former passivity: *“It was just really motivating to have the feeling ‘okay*,* I*,* I can actually do something by fasting […].’”* (Participant 4, aged 41, 1st fasting, Pos.42).

Against the backdrop of increased inner balance and moments of peace described above, participants also reflected on how fasting provided an opportunity to rethink and re-evaluate their relationship with their desire to conceive, including past experiences, their current situation, and future perspectives. Most participants were curious about their own and the study’s outcomes, while some gained hope for future fertility treatments after study completion. One participant became pregnant after or during fasting, which reinforced her own subjective belief in its benefits to fertility and shifted her focus on her future motherhood. Another participant reflected on the emotional toll that her desire to conceive had taken on her, becoming more aware of this burden during her fasting period. Some participants described moving beyond acknowledgement and towards acceptance. These descriptions highlighted how fasting helped them to let go of their intense focus on having children. One stated making peace with the possibility of it never happening: *“And what it actually did is that I (pause) am more relaxed regarding the fact that it might not work out. […] I feel more joyful and open again […] There’s more peace now. […] it will happen or it won’t happen […]”* (Participant 7, aged 39, 1st fasting, Pos.92).

### Challenges during fasting

Almost all participants described fasting as an acceptable intervention, with all but one adhering to the study’s fasting protocol. The participant who broke her fast prematurely did so after six days, citing an important business appointment as the reason: *“I didn’t want to go into the work meeting while fasting. […] Because I noticed I couldn’t concentrate that well while fasting.”* (Participant 5, aged 43, 1st fasting, Pos.12).

Despite the acceptability of the fasting protocol and regimen, all participants faced some fasting challenges to varying degrees. While half described their experience as relatively easy, the other half reported having difficulties: *“Yeah*,* it was just (pause) I’d say a necessary evil.”* (Participant 2, aged 32, 1st fasting, Pos.24). The distinction between frequently experienced but manageable challenges and those that were more difficult to manage provides insight into which challenges participants were able to tolerate and which posed greater difficulties.

Among the challenges that were frequently experienced and described as mostly manageable, all participants reported fatigue. Dissatisfaction with the taste of fasting meals was another common challenge, with participants describing them as tasteless, unpleasant in flavour, or monotonous. More difficult to manage were mood fluctuations, including moodiness and emotional instability. Some participants attributed these experiences to the absence of eating, which had otherwise been a source of enjoyment, and perceived the loss of this source of enjoyment as negatively affecting their mood. Most participants also reported food cravings, which were predominantly experienced as manageable. Cravings occurred more frequently than actual hunger. For some, hunger and cravings became more difficult to manage when surrounded by others who were eating. In such situations, some participants found shared meals particularly difficult to enjoy, as they heightened their sense of deprivation and prompted them to withdraw from social situations that often revolved around food. Conversely, some participants experienced their own social withdrawal as equally difficult. Notably, many believed fasting would have been easier in a group setting, underscoring the desire for a shared and food-free experience.

Fear of unexpected physical side effects, such as dizziness, and a feeling of being overwhelmed by these symptoms were particularly distressing for one participant. Some felt their workload hindered fasting, as the demands left little room for adjusting and slowing routines or adopting new habits. Several therefore expressed a desire for more leisure time to be built in future fasting plans. Furthermore, the challenges described above collectively contributed to impaired psychological wellbeing.

Challenges encountered are described in detail in an Additional file (see Additional file 5). The way that participants addressed, coped and contended with the challenges they faced provides important insights about study participant retention and individual risk-benefit calculation.

### Strategies for facing challenges and sustaining the fast

The variety of challenge types and intensities required participants to develop coping strategies for managing them. Resources were also reported to play an essential role in addressing these challenges, with motivation and a supportive environment being particularly important.

The explicitly stated primary motivation emphasized by participants was the desire to improve their chances of pregnancy through fasting and to enhance their overall health, including weight loss. 

*“[…] that it might work better with the desire to have children, that it helped eNORmously. Even if it was maybe only subconscious.”* (Participant 6, aged 37, 1st fasting, Pos.102) Knowing their reasons for fasting helped participants sustain their commitment even during challenging periods. While the initial motivation for fasting sparked their interest in participating and remained important throughout the fasting period, it was increasingly complemented by a growing sense of responsibility to contribute meaningfully to scientific research. Participants described valuing the opportunity to contribute to research and feeling a sense of responsibility toward the study. *“So I thought*,* ‘Wow*,* just go ahead and eat something on day three*,*’ but I knew*,* no*,* no*,* there are some people who are really COUNTING on me to stick with this*,* and that totally mOTIvated me. Just knowing that this was part of a study. If it HADN’T been for that*,* I WOULDN’T have lasted the full seven days.”* (Participant 3, aged 44, 2nd fasting, Pos.10).

A supportive environment, both professionally and personally, was described as a sustaining benefit. Support from the dietitian was considered essential, particularly for those fasting for the first time. *“If I had been alone*,* I think I would have quit […] and the measures that were suggested to me [by the dietitian] really helped*,* but I wouldn’t have thought of them myself*,* and I wouldn’t have had the energy to figure out the right measures.”* (Participant 7, aged 39, 1st fasting, Pos.50) All participants appreciated their interactions with the dietitian and the availability of additional support. Most participants who sought additional dietitian support did so due to physical complaints and felt reassured by the prompt assistance, tailored recommendations, and emotional support. Even those who did not seek help found comfort in knowing that support was available if needed. Some participants also perceived the dietitian’s calls as a form of compliance checks, which encouraged them to adhere to the study protocol. Partners’ encouragement was another important source of support, particularly when it involved adapting their eating habits: One participant highlighted her partner’s participation in fasting as a major source of support, while others appreciated their partners’ thoughtful eating habits.

Participants also described ways of individually addressing the challenges posed by fasting: They managed dissatisfaction with fasting meals by adjusting seasonings, using a juicer, or substituting brews and juices. Seeking distraction was also mentioned as helpful, whether through work, family, sleep, meal preparation, or spending time in nature. Most participants found that maintaining a calmer routine was beneficial, especially during periods of fatigue. Participants’ experiences suggest that the ability to adapt daily routines and workload to the fasting period may facilitate its manageability. Those with flexible jobs were able to adjust their workload accordingly, while many scheduled fasting during less demanding periods to make the process easier. Despite an optional longer fasting protocol, all participants initially chose or pared down their fasting routine to the minimum seven-day fast in order to better cope with competing challenges. These challenges included upcoming commitments, struggles with experienced side effects, lowered motivation, or self-doubt.

An Additional file outlines important factors described by participants for successful fasting (see Additional file 6).

## Discussion

### Summary of the results

Overall, the prolonged fluid-only fasting programme seemed to be well accepted by participants experiencing infertility. Participants reported both benefits and challenges across emotional, physical, and social aspects. Motivation, individual coping strategies, and dietitian support helped them overcome difficulties. The primary motivation for most participants was the hope of potentially benefiting from fasting regarding their fertility. After fasting, participants´ attitudes toward having children varied, ranging from increased hope for fertility treatment to a reduced focus on parenthood.

### Strengths and limitations

To our knowledge, this exploratory qualitative study is the first in this emerging field to explore how women experiencing infertility experience prolonged fluid-only fasting. The study includes a relatively small sample (*n* = 8), consisting predominantly of highly educated, employed, middle-class German women. However, recruitment challenges in the RCT limited our ability to recruit a larger and more heterogeneous sample, as participant inclusion depended on overall trial recruitment. Therefore, theoretical saturation cannot be assumed and the findings should be interpreted with caution regarding their transferability to women with different social and cultural backgrounds. A larger and more diverse sample, with greater variation in educational and socioeconomic backgrounds, migration and cultural backgrounds, and fasting experiences, might have revealed different experiences and ways of integrating the fasting intervention into everyday life. For example, participants engaged in physically demanding work might have faced different challenges in maintaining the fasting regimen than those with predominantly sedentary occupations. Previous familiarity with fasting within cultural or family contexts might have provided additional sources of support or shaped expectations regarding the fasting experience. Similarly, differences in cultural expectations surrounding motherhood and childbearing might have influenced the experience of infertility-related stress and, consequently, how participants perceived and coped with fasting. Only one participant in our sample discontinued the fasting protocol prematurely. Therefore, the perspectives of women who may have discontinued the protocol, as well as their potential reasons for doing so, are likely underrepresented. Accordingly, the experiences described in this study may not fully reflect those of women with different social, cultural, reproductive, or experiential backgrounds.

Furthermore, restrictions imposed by the COVID-19 pandemic led us to conduct online interviews, thereby limiting the interpretation of the participants´ body language and might have impacted interpersonal dynamics.

Another potential limitation concerns the researchers’ backgrounds and perspectives. Some members of the team have research and, in some cases, clinical experience in integrative and fasting medicine and therefore brought both professional expertise and a personal interest in the potential benefits of the fasting approach. This proximity to the field may have facilitated an informed and context-sensitive approach to the data but may also have introduced preconceptions regarding fasting and its potential benefits. In particular, participants may have been inclined to provide socially desirable responses, while researchers may have been more likely to interpret statements in ways consistent with their professional expectations. At the same time, the interviewer was a relatively young researcher with no prior experience in qualitative research, reproductive challenges, or fasting research. While the lack of qualitative research experience may have influenced the interview process and interpersonal dynamics, her lack of specific expertise in fasting also meant that she approached the interviews without established assumptions about fasting and its potential effects. This may have facilitated an open exploration of participants’ experiences.

These potential influences were addressed through ongoing reflexive discussion within the research team and investigator triangulation during coding and interpretation. Preliminary findings and interpretations were discussed with team members, including researchers who had not previously conducted research on fasting, as well as in a qualitative research colloquium involving researchers with diverse thematic and methodological backgrounds. Divergent cases and alternative interpretations were actively sought and discussed. We therefore consider the researchers’ diverse backgrounds and perspectives to have been both a potential source of bias and a resource: the clinical and research expertise provided contextual knowledge of fasting, while perspectives from researchers without specific expertise in fasting and from qualitative researchers with different thematic backgrounds helped to challenge potential preconceptions. Nevertheless, the influence of the researchers’ professional backgrounds and views on fasting cannot be fully excluded and should be considered when interpreting the findings.

### Interpretation

#### High adherence suggests acceptability

All but one participant adhered to the fasting protocol, suggesting that the fasting regimen was acceptable to most participants, consistent with previous studies reporting high adherence rates during fasting [34, 48, 49]. The participant who discontinued fasting described the overall experience as easy, but discontinued due to an upcoming appointment, suggesting that careful timing around important personal commitments may be important for maintaining adherence.

#### What makes fasting difficult? Factors shaping the perceived difficulty of fasting

The fasting intervention was described as difficult by half of the participants, although none of them discontinued it. Acceptability should therefore not be equated with ease, raising the question of what factors made fasting particularly difficult. Previous research suggests that initial fasting experiences may be associated with more negative psychological effects than fasting among more experienced fasters. In one study, participants without previous fasting experience reported greater appetite, more negative mood states, higher stress and fatigue, and fewer recovery resources than experienced fasters [50]. This may be particularly relevant to our findings, as most participants were fasting for the first time. For first-time fasters, unfamiliarity with the physical and psychological sensations of fasting may make challenges such as fatigue, cravings, or fluctuations in mood more difficult to anticipate and manage. At the same time, individual responses to the same condition may differ substantially, which may also help explain why participants experienced the identical fasting conditions as more or less challenging [51, 52]. Although the intervention was standardized in terms of fasting duration, dietitian support, and materials, participants’ experiences were shaped by the individual and contextual circumstances in which fasting took place. Moreover, the challenges described by participants were not necessarily independent of one another but could interact and compound each other, potentially contributing to broader psychosocial consequences of fasting-related disruptions to everyday life.

For example, participants cited workload and a resulting lack of leisure time as major challenges. As shown in the presentation of positive fasting effects, many beneficial practices also required sufficient time to be incorporated into the fasting routine. Greater availability of leisure time may therefore not only reduce the challenges posed by work obligations during fasting but also facilitate the integration of beneficial practices, potentially making the fasting experience more manageable and positively perceived. Some participants addressed this challenge by reducing their workload. This underscores the importance of scheduling fasting during periods with fewer work-related demands and considering the extent to which participants are able to adapt their daily routines to the demands of the intervention.

Another example was the interaction between food-related cravings, dissatisfaction with fasting meals, and social interactions. Cravings, reinforced by dissatisfaction with fasting meals, made food-related environments and, consequently, social interactions more challenging to navigate. Avoiding such environments may serve as a coping strategy for managing cravings and feelings of deprivation. However, this strategy may have further consequences: by limiting participation in social interactions that often take place around food, participants may experience social withdrawal and a sense of missing out, while also losing an important source of social well-being. These interconnected challenges may therefore contribute to feelings of emotional instability and low mood. This may be particularly relevant in the context of infertility, where social withdrawal and reduced social participation have been described as aspects of the psychosocial burden associated with infertility [53, 54].

The social consequences of food-related restrictions can be understood in light of the broader social and cultural significance of food and eating practices [55–57]. Eating together, or commensality, is not merely a way of consuming food but can serve important functions in maintaining social relationships and a sense of belonging [56, 58]. From this perspective, fasting temporarily alters not only nutritional practices but also established forms of social participation. The challenges described by participants may therefore reflect a disruption of commensal practices rather than difficulties related to food deprivation alone. This broader perspective helps contextualize why food-related restrictions may affect the social experience of fasting beyond the immediate difficulties of hunger or cravings. While actively creating social moments outside meals was not mentioned as a coping strategy, the assumption that fasting in a group setting would have been easier suggests that participants may have perceived social connection as a potential way of managing these challenges. Future fasting interventions could therefore consider opportunities for social connection, for example through group-based fasting or social activities outside food-centred settings.

Beyond its social function, food also contributes to the sensory and pleasurable dimensions of everyday life [57]. The importance of taste and enjoyment during fasting is supported by previous studies, which identify taste dissatisfaction as a challenge and report dissatisfaction with fasting meals as a reason for discontinuation [59, 60]. Preserving opportunities for taste and enjoyment, whether through fasting meals or other sources of pleasure, may therefore be relevant not only for adherence but also for a more positive overall fasting experience.

#### The dynamic nature of the fasting experience

While some of the positive fasting effects reported by participants may initially appear to contrast with the challenges they experienced, these findings are not necessarily contradictory. A prolonged fasting experience may involve both inter-individual variation and intra-individual changes over time. The fasting phases described by Brunnhuber and Somburg capture this temporal variation and highlight how the psychological experience of fasting may change across different phases [61]. The positive experiences reported in the present study are also consistent with findings from previous studies of prolonged fasting [32–34, 48, 62, 63]. Rather than being merely incidental aspects of the fasting experience, these positive experiences may be particularly relevant in the context of an unfulfilled desire for children.

#### From coping to self-efficacy and acceptance

The challenges encountered during fasting required the development and use of coping strategies to maintain the fast. Successfully managing these challenges may have strengthened self-efficacy beyond the sense of “I can actively participate in my treatment” towards “I can cope with difficult situations.” This may have increased participants’ confidence in their ability to deal with the uncertainties and challenges of the fertility journey, including the possibility that their desire for children might ultimately remain unfulfilled. Such acceptance could shift the experience of an unfulfilled desire for children away from feelings of helplessness and failure towards a more self-determined approach to the fertility journey and the possibility of alternative life plans without children. Against this backdrop, the premature termination of the fast by one participant can also be understood as a self-determined decision. In the context of infertility, where social expectations surrounding parenthood are particularly salient, the ability to set personal limits and make autonomous decisions may itself represent an important resource.

#### Active engagement as a source of motivation and agency

While personal benefits were important for initiating the fast, other motivators, such as a sense of responsibility towards the study and the wish to contribute to research, became increasingly important during fasting. Such study-related motivators have also been described in previous research and may not be present to the same extent when fasting interventions are offered in routine clinical practice [64]. They should therefore be considered when interpreting adherence in research settings.

The importance of active engagement in fertility care provides a further context for understanding participants’ motivation to undertake fasting. The desire to actively participate in treatment is frequently cited as a motivation for using traditional and integrative medicine among couples experiencing infertility and was also an important motivation in the present study [35, 60, 65]. The motivation to actively influence fertility and health can be understood in light of Bury’s concept of agency and control in the face of illness [11]. Participants’ descriptions of fasting as an opportunity to actively do something for their fertility and health reflect this process of regaining a sense of agency and control, and were an important resource supporting both fasting initiation and adherence. This active engagement may have been particularly meaningful in the context of infertility, where uncertainty and a perceived lack of control are well documented [9, 12–14].

#### Hope and pressure: the ambivalence of having options

While Bury’s concept highlights the role of active engagement in restoring agency and control, Franklin’s concept of “hope technologies” draws attention to another dimension of such engagement: its capacity to sustain hope in the face of reproductive uncertainty. Franklin describes in-vitro-fertilisation as a practice that can sustain hope for achieving parenthood even when the outcome remains uncertain https://doi.org/10.4324/9781003284499. Although fasting is not a reproductive technology in the conventional sense, participants perceived it as an opportunity to actively contribute to their chances of becoming pregnant. The importance participants placed on actively doing something may therefore reflect not only a desire to exercise agency and control, but also a way of sustaining reproductive hope. This perspective points to a potential ambivalence of active engagement: the same practices that sustain hope and provide a sense of agency may also create an expectation to continue trying as long as further options remain https://doi.org/10.4324/9781003284499. For some participants, the challenging nature of fasting may therefore have contributed to acceptance by reinforcing the sense that they had “tried everything” in order to conceive. This raises the possibility that acceptance may, for some individuals, be easier to reach after they feel that they have exhausted the available options.

#### Renegotiating reproductive identity

These processes of active engagement, coping, and growing acceptance may also help explain the changes in reproductive identity reported by participants. Thus, fasting did not appear to lead to a uniform change in reproductive identity. Rather, it provided an opportunity for participants to reflect on and renegotiate their reproductive expectations, identities, and imagined futures.

Bury’s framework is relevant here not only in relation to agency and control, but also to the disruption of identity and future expectations [11]. Research on infertility has highlighted the impact of reproductive disruption on identity and the emotional work involved in managing changes in expectations and future possibilities [66]. Ussher et al. further conceptualize infertility following cancer as a threat to the expected life course, highlighting how reproductive disruption can challenge established identities, gendered expectations, and imagined futures [8]. From this perspective, the changes in reproductive identity reported by participants can be understood as part of a broader process of renegotiating disrupted biographical expectations.

The renegotiation of reproductive identity was accompanied by changes in how participants related to their bodies and themselves. While infertility may be accompanied by a negative body image and a sense that one’s body is not “working correctly,” fasting appeared to provide an opportunity to develop a more caring relationship with the body and with oneself [67]. For many participants, this was reflected in a kinder way of treating themselves, including actively slowing down their routines, feeling pride in caring for their bodies, experiencing enjoyment, engaging in reflection, and turning their attention inward.

The reported changes in participants’ relationships with their bodies and themselves may be particularly relevant in light of Brunnhuber and Somburg’s conceptualisation of the psychological processes involved in fasting [61]. Their description of an initial phase of adjustment followed by increasing self-efficacy and des-identification resonates with several aspects of participants’ accounts. Here, des-identification refers to a process of gradually distancing oneself from experiences with which one would ordinarily identify oneself, beginning with bodily processes and sensations and potentially extending to thoughts, affects, autobiographical experiences, life goals, and meaning-making [61]. This process may be reflected in the present study through participants’ changing relationships with their bodies and the development and renegotiation of their reproductive identities. According to Brunnhuber and Somburg, this psychological process during fasting may ultimately extend to the ego and its functions, opening up a different level of experience associated with transpersonal affects such as gratitude, humility, forgiveness, compassion, and a sense of connectedness with others [61]. This conceptualisation resonates with participants’ reports of increased calmness, inner balance, and mindfulness during fasting. In this sense, fasting may represent more than a form of caloric restriction with physiological effects; it may also have served as a structured behavioural and emotional practice that provided a framework for changes in routines, self-perception, and emotional experience.

### Implications for future research and practice

Regarding future research perspectives, this study provides insights that may inform the planning and design of prolonged fasting interventions in the context of infertility. Although this study focused on women experiencing infertility, some of the practical considerations identified may also be relevant to prolonged fasting interventions in other research or clinical settings. Key considerations include comprehensive counselling to determine an appropriate fasting period, close support from experienced professionals with good availability, and attention to the palatability and variety of fasting meals. In addition, supporting individual resources, particularly through social support and opportunities for social connection, may help participants integrate fasting into their everyday lives. Future research should examine whether and how these factors influence the acceptability and manageability of prolonged fasting across different populations and settings. Additionally, quantitative studies are needed to determine whether the perceived hope for an increased likelihood of pregnancy is supported by measurable fertility outcomes. 

## Conclusion

Medically supervised prolonged fluid-only fasting was not only perceived as acceptable by women experiencing infertility in an outpatient setting, but participants described several perceived benefits, including a sense of inner balance, enhanced self-care, an increased feeling of agency in their fertility journey, and renewed hope. Although participants reported challenges such as fatigue, cravings, and social restrictions, these were often manageable with appropriate support.

## Supplementary Information

Supplementary Material 1.

Supplementary Material 2.

Supplementary Material 3.

Supplementary Material 4.

Supplementary Material 5.

Supplementary Material 6.

## Data Availability

The datasets generated and/or analysed during the current study are not publicly available due to potential patient identifiers in datasets (Verbatim responses or transcripts) but are available from the corresponding author on reasonable request.
