# Supplementary material for: Women’s experiences of a medically supervised fasting intervention for infertility: a qualitative study
Source: BMC Womens Health. 2026 Oct 9;26:511. doi: 10.1186/s12905-026-04978-y (PMC13653389; doi:10.1186/s12905-026-04978-y)
Supplement: Supplementary file 3 [file 12905_2026_4978_MOESM3_ESM.docx]

### Additional file 3: Interview Guide

| **Narrative Prompt**  **"Go ahead and tell me your story. You can’t tell too much."** | **Content-Related Aspect**  Already mentioned? If not, bring it up at the right moment with the appropriate phrasing. | **Specific / Follow-up Questions** | **Ways to maintain narrative flow** |
| --- | --- | --- | --- |
| How did you feel while fasting?  And during the period in general? | - What went well? Why? - What didn’t go well? Why? How did you handle it? - Well-being - Changes: Has anything changes since you started fasting therapy regarding …? - Motivation - How did you feel? | - In your opinion, what was important in order to succeed in fasting? / What do you think would have been important to succeed in fasting? - Would you do anything differently if you fasted again? - Is there anything else you would like to say about fasting? | - Non-verbal encouragement (active listening, nodding, etc.) - Can you tell me a little more about that? - Does anything else come to mind? - And then? How did it continue? - Can you describe an example of that? - Put yourself back in situation X for a moment. - What does X mean to you? - What do you understand by X? - How did that come about? - You already touched on this… please elaborate on this aspect in more detail." - Let me check if we’ve forgotten anything… - What do you mean by that? - Would it be possible to describe in more detail what you mean? - Repeating: "Did I understand that correctly? - Is there more to that? - What else? |
| How did you find the fasting support? | - Facilitation - Would have preferred it differently - Questionnaires | - Would you like to share any feedback about the fasting support? |  |
| How did you feel when it was clear that you were assigned to the fasting group? | - Motivation |  |  |
| You are participating in the study because of your desire to have children: Would you like to share anything about that? | - Changes or effects - Partnership - Self-image - Stress - Acceptance as therapy option? |  |  |
| We notice in the study that many women who learn about the study in fertility clinics decide against participating. Do you have any idea why that might be? |  |  |  |
