# Supplementary material for: Women’s experiences of a medically supervised fasting intervention for infertility: a qualitative study
Source: BMC Womens Health. 2026 Oct 9;26:511. doi: 10.1186/s12905-026-04978-y (PMC13653389; doi:10.1186/s12905-026-04978-y)
Supplement: Supplementary file 4 [file 12905_2026_4978_MOESM4_ESM.docx]

### Additional file 4: Positive fasting effects

| **Category** | **Main finding** | **Pertinent quote** |
| --- | --- | --- |
| **Beneficial fasting practices** | The fasting period was often perceived as an opportunity to incorporate moments of peace, physical activity, and self-care, facilitated by the absence of regular mealtimes. Measures and activities that enhanced participants’ well-being were often inspired by recommendations from the study team. These included exercises, periods of relaxation, bowel cleansing, and liver compresses. Maintaining or returning to usual beneficial routines was also perceived positively and was described as being more strongly valued in the context of increased inner balance and mental altertness. | *“But during that time, I just took everything more calmly. But maybe that’s also because deep down, I wanted that. You know? A bit of (pause) going inward, reflecting, you know?”*  (Participant 8, aged 39, 2^nd^ fasting, Pos.90)  *“And at some point, I noticed that it’s nicer for me and good for me when we sit down TOGETHER and eat TOGETHER. It’s just that I spoon my vegetable broth while [my partner] eats his food. But it still keeps that meal-time togetherness. And at some point, it worked out quite well.”*  (Participant 1, aged 30, 1^st^ fasting, Pos.22) |
| **Positive states of mind** | Positive feelings and moods were categorized under positive states of mind, including a sense of well-being, improved mood, mental alertness, inner balance, pride in caring for their bodies, and enjoyment of fasting meals—marked by appreciation for the preparation, flavours, and variety of homemade juices and broths. | *“Yes, as I said, I was much more relaxed than usual. Also much more balanced. […] It’s often the case that my mood fluctuates a lot or I react strongly to things that annoy me. And that was much more balanced.”*  (Participant 7, aged 39, 1^st^ fasting, Pos.24)  *“And I really had, like, a razor-sharp mind. I didn’t forget anything. I got everything done that I wanted to. I was very fast, very clear in my communication.”*  (Participant 3, aged 44, 2^nd^ fasting, Pos.52)  *“[…] beforehand, I always had this little slump around midday, but I didn’t have that anymore. And I was actually already alert in the morning. I got an incredible amount done. And yet, I didn’t feel exhausted.”*  (Participant 4, aged 41, 1^st^ fasting, Pos.19-20)  *“I either took a nice broth with me or made a juice and so on. But I consciously enjoyed that. I didn’t always, but sometimes I even SCOOPED it […]. Or drank it sip by sip. (pause) It was a highlight.”*  (Participant 6, aged 37, 1^st^ fasting, Pos.24) |
| **Physical wellness** | A general sense of physical wellness, attributed to a feeling of lightness from weight loss and improvements in other health issues, such as hip-pain, digestive problems and low energy, typically experienced after meals. Also, boost in energy and feeling of alertness from the morning onward. | *“But once I was in it, it also felt really good. Because I noticed (pause) it’s just GOOD for me. It’s good for my body as well. I also noticed that the bloating, I have a bit of trouble with irritable bowel, the bloating also decreased.”*  (Participant 5, aged 43, 1^st^ fasting, Pos.18)  *“It was just nice to feel a bit lighter!”*  (Participant 8, aged 39, 2^nd^ fasting, Pos.98) |
| **Concerns didn’t materialise and absence of challenges** | Positive experience when expected hunger or moodiness did not occur or after overcoming previous challenges. | *“So I wasn’t constantly hungry the whole time. And I thought that was good. Because I really thought I would (pause) be walking around with hunger all the time, yeah.”*  (Participant 4, aged 41, 1^st^ fasting, Pos.10) |
