# Supplementary material for: Women’s experiences of a medically supervised fasting intervention for infertility: a qualitative study
Source: BMC Womens Health. 2026 Oct 9;26:511. doi: 10.1186/s12905-026-04978-y (PMC13653389; doi:10.1186/s12905-026-04978-y)
Supplement: Supplementary file 5 [file 12905_2026_4978_MOESM5_ESM.docx]

### Additional file 5: Frequent and difficult challenges during fasting

| **Category** | **Main findings** | **Pertinent quote** |
| --- | --- | --- |
| **Physical challenges** | Physical challenges include physical complaints that interviewees attributed to fasting. A frequently reported challenge was fatigue, characterized by low energy, weakness, and inertia. Hunger was reported less often but experienced as difficult challenge when present. Hunger describes the physical longing for food, while appetite and cravings are listed among psychological challenges. | *“Otherwise, at home, I sometimes do sports as well […] But I didn’t do that during that time either because I just didn’t feel fit enough to think, ‘Yes, I’ll do that now.’ I think I still did on the first day, but after that, not anymore. […] Even for walks […] I had to push myself a little just to get moving at all. Like I said, I just stayed on the sofa or something.”*  (Participant 2, aged 32, 1^st^ fasting, Pos.30)  *“When you start feeling a bit more sluggish or, let’s say, tired, right? Or I just notice it when I simply don’t have the strength for big things.”*  (Participant 8, aged 39, 2^nd^ fasting, Pos.90)  *“I just had this constant, really REAL feeling of hunger and like, ‘Oh no, not again.’”*  (Participant 3, aged 44, 2^nd^ fasting, Pos.18)  *“The only thing that really bothered me was the hunger […].”*  (Participant 7, aged 39, 1^st^ fasting, Pos.12) |
| **Challenges in the realisation of everyday fasting** | Challenges in implementing everyday fasting included difficulties in adapting their routines to fasting or following the study team’s instructions and recommendations. A frequently reported challenge was dissatisfaction with the fasting meals, described as tastelessness, unpleasant flavours, or monotony. Some interviewees also found it particularly difficult to manage fasting alongside a continuous high workload. | *“That’s just the juice and the broth. I don’t know if I did something wrong, but it just did not taste good to me AT ALL.”*  (Participant 2, aged 32, 1^st^ fasting, Pos.16)  *“But still, [a juice] naturally has much less (pause) flavour than a full meal. And (pause) at some point, that made me lose interest a little. […] That sense of ENJOYMENT kind of got lost.”*  (Participant 1, aged 30, 1^st^ fasting, Pos.24)  *“Simply because of my job, since I was often traveling, it became more or less easy to integrate depending on the situation. And (pause) at the beginning, it was actually quite difficult at times.”*  (Participant 1, aged 30, 1^st^ fasting, Pos.10) |
| **social withdrawal** | Social withdrawal refers to a loss of enjoyment in social gatherings and a reduction in social interactions, a challenge experienced by some interviewees who found it particularly difficult. | *“At times, I really thought that FASTING almost completely sucks out all joy in life. Because somehow—well, I think it has to do with the fact that meals strongly structure the day (pause) and are often linked to social interactions, and all of that just falls away at first […]”* (Participant 3, aged 44, 2^nd^ fasting, Pos.8)  *“And (pause) then you cook with your family, then there’s the Christmas market. So you just go along. And everyone is eating—except you. (laughs) (pause) Maybe not the most helpful situation.”*  (Participant 8, aged 39, 2^nd^ fasting, Pos.126) |
| **Psychological challenges** | Psychological challenges refer to emotionally or mentally difficult experiences. Cravings were frequently reported but were not described as particularly difficult. Other psychological challenges, however, were perceived as more difficult, including emotional instability, low mood, and loss of enjoyment. Fear and sense of overwhelming from unexpected physical side-effects of fasting, such as dizziness, stomach pain, or back pain, were perceived as difficulty by one interviewee. Furthermore, the challenges described above led to impaired psychological wellbeing as well. | *“[…] I had such an uncontrollable craving for plain tofu. And I still do. It’s just so CRAZY. And then just a crispbread with plain tofu and a tomato on top. That’s REALLY the best.”*  (Participant 7, aged 39, 1^st^ fasting, Pos.34)  *“Because I noticed that in the evenings, my mood was often more irritable, and I was a bit more sensitive and (pause) yeah. That wasn’t so nice and (pause) over time, it was also exhausting.”*  (Participant 1, aged 30, 1^st^ fasting, Pos.2)  *“Because at times, things happened that really SHOCKED me, and I thought, ‘Okay, is this normal or not, and what can I do?’”*  (Participant 7, aged 39, 1^st^ fasting, Pos.6) |
