# Supplementary material for: Women’s experiences of a medically supervised fasting intervention for infertility: a qualitative study
Source: BMC Womens Health. 2026 Oct 9;26:511. doi: 10.1186/s12905-026-04978-y (PMC13653389; doi:10.1186/s12905-026-04978-y)
Supplement: Supplementary file 6 [file 12905_2026_4978_MOESM6_ESM.docx]

### Additional file 6: Overview of resources and coping mechanisms

| **Category** | **Main findings** | **Pertinent quote** |
| --- | --- | --- |
| **Motivation** | Motivation refers to the reasons why participants chose to start and continue fasting.  Initially, interviewees mainly hoped to improve their fertility and their overall health. During fasting, the contribution to research and a sense of responsibility toward the study, gained importance. | *“[…] that it might work better with the desire to have children, that it helped eNORmously. Even if it was maybe only subconscious.”*  (Participant 6, aged 37, 1^st^ fasting, Pos.102)  *“So I thought, ‘Wow, just go ahead and eat something on day three,’ but I knew, no, no, there are some people who are really COUNTING on me to stick with this, and that totally mOTIvated me. Just knowing that this was part of a study. If it HADN’T been for that, I WOULDN’T have lasted the full seven days.”*  (Participant 3, aged 44, 2^nd^ fasting, Pos.10)  *“To make it through in the end, the important thing wasn’t really […] how it would affect the (pause)/ the desire to have children. That was somehow secondary. What was really primary was the thought, ‘I’m doing this now, and I’m going to see it through. I’ve committed to do this for seven days, so I’ll do it.’”*  (Participant 3, aged 44, 2^nd^ fasting, Pos.46) |
| **Support system** | The support system consisted of actions or measures of interviewees environment easing the fasting process.  Especially professional support was described as crucial. All participants appreciated their interactions with the dietitian and the availability of additional support. Some also valued their partner’s encouragement, particularly when it involved adjustments to their eating habits. | *“Yes, being supported through the study gives you more determination compared to doing it alone and without any support.”*  (Participant 5, aged 43, 1^st^ fasting, Pos.60)  *“I had never fasted before, and I don’t think I would have ever done it on my own. So I found it really helpful to have support, and the nutritionist, whom I could contact at any time, was also a great help to me.”* (Participant 7, aged 39, 1^st^ fasting, Pos.4)  *“And that really helped me because I always knew what to expect and also knew that if I wasn’t feeling well or had circulatory issues, I had someone to call and ask, ‘What can I do now?’”*  (Participant 4, aged 41, 1^st^ fasting, Pos.38)  *“If I had been alone, I think I would have quit […] and the measures that were suggested to me really helped, but I wouldn’t have thought of them myself, and I wouldn’t have had the energy to figure out the right measures because, of course, it’s BETTER if you’re told quickly rather than having to search through a thousand things on the internet or something.”*  (Participant 7, aged 39, 1^st^ fasting, Pos.50)  *“My husband didn’t participate, but he was such a great companion. He also ate very, very healthily and reduced his food intake, but he didn’t fast completely. And he was a really good source of support.”*  (Participant 6, aged 37, 1^st^ fasting, Pos.2) |
| **Individual coping strategies** | Reactions that interviewees actively took in response to challenges are presented under individual coping strategies. The main coping strategies included adjusting fasting meals, using distractions, seeking dietitian support, establishing a calmer routine and shortening the fasting period to a minimum. | *“[…] at the same time (pause) I noticed that through work and daily activities, it was also EASIER because I simply had other things to do.”*  (Participant 1, aged 30, 1^st^ fasting, Pos.52)  *“I also always turned in relatively early because I thought, ‘Ah, now you can save yourself an hour with the growling stomach and just go to sleep.’”*  (Participant 3, aged 44, 2^nd^ fasting, Pos.62)  *“[…] I was extremely weak the entire time. […] I couldn’t even imagine doing any exercise. […] Luckily, I was able to spontaneously arrange with my employer to work from home. […] I started on Friday and had the day off, so I could basically spend the first three days of fasting (pause) without any obligations. That was really good.”*  (Participant 7, aged 39, 1^st^ fasting, Pos.8)  *“[…] it was always said that the first few days are very difficult. For me, it was more like the last two days. That’s why I didn’t fast for ten days (pause) It also had to do with different things, like having challenging shifts ahead of me and other appointments.”*  (Participant 6, aged 37, 1^st^ fasting, Pos.2) |
